# Different Schiff Bases—Structure, Importance and Classification

**DOI:** 10.3390/molecules27030787

**Published:** 2022-01-25

**Authors:** Edyta Raczuk, Barbara Dmochowska, Justyna Samaszko-Fiertek, Janusz Madaj

**Affiliations:** Carbohydrate Chemistry Group, Faculty of Chemistry, University of Gdańsk, Wita Stwosza 63, 80-308 Gdańsk, Poland; edyta.raczuk@phdstud.ug.edu.pl (E.R.); j.samaszko-fiertek@ug.edu.pl (J.S.-F.); janusz.madaj@ug.edu.pl (J.M.)

**Keywords:** Schiff bases, hydrazides, dihydrazides, hydrazones, hydrazide–hydrazones

## Abstract

Schiff bases are a vast group of compounds characterized by the presence of a double bond linking carbon and nitrogen atoms, the versatility of which is generated in the many ways to combine a variety of alkyl or aryl substituents. Compounds of this type are both found in nature and synthesized in the laboratory. For years, Schiff bases have been greatly inspiring to many chemists and biochemists. In this article, we attempt to present a new take on this group of compounds, underlining of the importance of various types of Schiff bases. Among the different types of compounds that can be classified as Schiff bases, we chose hydrazides, dihydrazides, hydrazones and mixed derivatives such as hydrazide–hydrazones. For these compounds, we presented the elements of their structure that allow them to be classified as Schiff bases. While hydrazones are typical examples of Schiff bases, including hydrazides among them may be surprising for some. In their case, this is possible due to the amide-iminol tautomerism. The carbon–nitrogen double bond present in the iminol tautomer is a typical element found in Schiff bases. In addition to the characteristics of the structure of these selected derivatives, and sometimes their classification, we presented selected literature items which, in our opinion, represent their importance in various fields well.

## 1. Introduction

The term Schiff’s base derives from the name of the German chemist Hugo Schiff, who, in 1864, was the first to describe the products resulting from the reaction of primary amines with carbonyl compounds [1].

**Figure 1:**
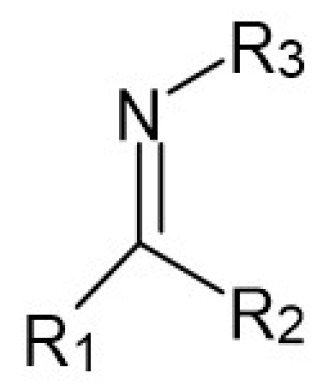
The specific structure fragment characteristic of Schiff bases, where R_1_, R_2_ and R_3_ are alkyl or (more often) aryl groups. R_1_ or/and R_2_ may also be hydrogen atoms.

Following the recommendation of IUPAC, Schiff bases are defined as chemical compounds (imines) bearing a hydrocarbyl group on the nitrogen atom R_2_C = NR′ (R′ ≠ H) (Figure 1). They are considered by many to be synonymous with azomethines [2].

**Figure 2:**
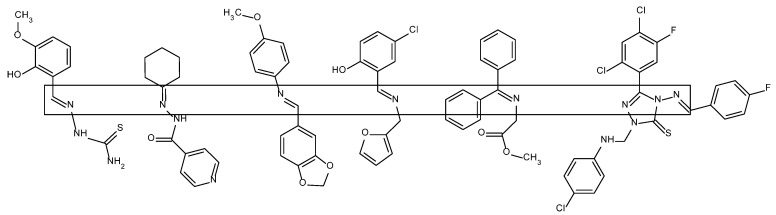
Examples of Schiff bases—the imine fragments are framed [12].

This common feature determines the ability of Schiff bases to form complexes with transition metal ions [3,4,5,6,7]. In these complexes, they function (like amines, amides and phosphines) as L-type ligands, that is, ligands containing two-electron donors, which do not undergo electron changes on their valence shells [8]. The complex formation takes place by coordinating the *d*-block metal ion by the electron-donating ligand atom and serves to modify the steric and electronic surrounding of the metal. As a consequence, this leads to the stabilization and regulation of the reactivity of the metal ion, which is especially useful for less stable ions at higher oxidation states [8,9]. Nitrogen, oxygen or sulfur atoms can participate in the coordination as donors. Multivalent Schiff base ligands (Figure 2) eagerly form complexes: bidentate and tridentate with Co(II), Ni(II) ions, as well as four-dentate, highly stabilizing metal ions at various oxidation states, starting with divalent Ni(II), Cu(II), Pd(II) or tetravalent V(IV), Ti(IV), up to uranium U(III,IV,V) [10,11]. The donor atoms (O, N, S) of bidentate ligands can occur, for example, in ON or NN sequences, tridentate ligands—ONO, NNN or ONS or NNS (where the sulfur atom comes from, for example, disulfide bridges) and in tetradentate ligands—NNNN, ONNO, NSNO [10,12,13].

Interest in the use of transition metal complexes with Schiff bases in medicine began to develop in the second half of the 19th century. Co(II), Ni(II), Cu(II) and Zn(II) complexes exhibit exceptional biological activity [14,15,16,17,18,19,20,21,22,23,24,25,26]. In recent years, there has been greater interest in the possibility of using metal ions such as Ag(I), Au(I) or Pt(II) in medicine [27,28]. Additionally, complexes of these metals with Schiff’s bases show interesting biological properties [29,30,31,32,33].

Schiff bases are called auxiliary ligands because they modulate the structure and reactivity of the transition metal ion in the center of the complex, while they do not undergo irreversible transformations themselves, unlike reactive ligands [8,9].

**Figure 3:**
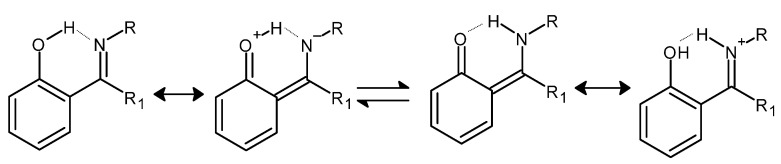
An example of the formation of intra-molecular hydrogen bonds by Schiff bases in keto-enol equilibrium together with resonance structures of the corresponding forms (R and R_1_ = alkyl groups) [34].

In the case of Schiff bases containing a benzene ring that is directly connected to an azomethine (imine) moiety, the presence of a hydroxyl group in the 2-position (*ortho*) to the moiety characteristic of Schiff bases may contribute to the formation of intramolecular resonance-stabilized hydrogen bonds (Figure 3), whereas their presence has a positive effect on the thermodynamic stability of the whole molecule [34].

Schiff bases have a number of applications as catalysts, including acid catalysts [33,35,36,37], reduction [38,39] or oxidation [40,41,42,43,44,45,46] catalysts.

Schiff bases are used, inter alia, in catalytic reactions, in crystal engineering, also as photo- or chemodetectors in biological systems (e.g., Al^3+^ ions in vivo) and most commonly, in medicine. Their most important medical applications include: antibacterial [47,48] and antifungal [49] (including anti-yeast) activity, antiviral [50,51], antitumor [52,53], anti-inflammatory [54], antipyretic, antimalarial [55], anticancer [56,57,58], anesthetic, oxytocin-imitating and oxytocin-inhibiting activity, as well as the selective inhibition of human tyrosine phosphatase 1B (PTP1B) or TCPTP and SHP-1 tyrosine phosphatases [12,34,59,60]. While there are indeed mentions of free ligands being more effective than their respective complexes [12], most often, it is the complexes of Schiff bases and metal ions that exhibit the strongest of the above-mentioned antimicrobial properties, creating favorable conditions for the penetration of microbial cell membranes by the metal ions they carry [12,61,62].

## 2. Hydrazides

### 2.1. Structure

**Figure 4:**
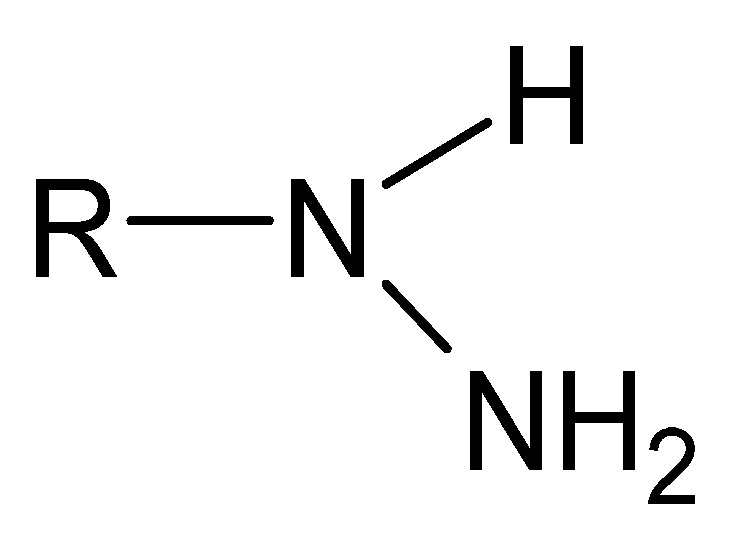
A moiety characteristic of hydrazides, where R is -C(=O)- or -(O=)S(=O)-.

A particular example of Schiff bases are hydrazides in their iminol tautomeric form. Hydrazides are a group of unique monosubstituted hydrazine derivatives that not only retain their specific -NH-NH- nitrogen bridge but also contain a carbonyl or sulfonyl group linked directly to one of the nitrogen atoms (Figure 4). The distinctive terminally occurring hydrazide moiety is as follows: R-NH-NH_2_ [62].

It should be noted that the illustrated hydrazide moiety may be partly analogous to the characteristic amide (peptide) moiety: (O=)C-NH-, the presence of which makes one think of hydrazides as potential peptidomimetics. An additional consequence of such a close proximity to oxygen and nitrogen atoms endowed with lone electron pairs, forming a torsion angle with the carbonyl atom and the hydrogen atom connected to the nitrogen atom, respectively, is the possibility of the migration of the double bond between the carbon and nitrogen atoms, with the formation of the hydroxyl group by the carbonyl oxygen atom [63,64,65].

**Figure 5:**
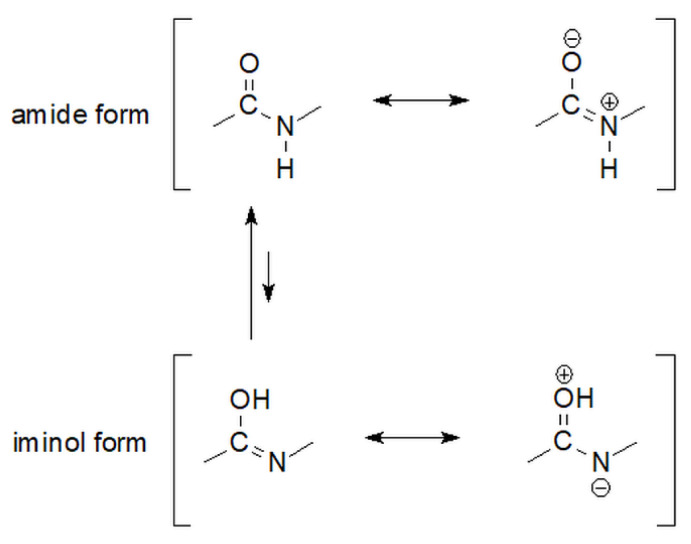
The equilibrium of amide-iminol tautomers of carbonyl hydrazides [66].

The above-mentioned amide bond tautomerism characteristic of peptides also occurs in hydrazides (Figure 5).

The high rotation barrier around the C=N bond limits conformational changes within the molecule and results in the existence of iminol forms of hydrazides, similar to the existence of amides, in the form of *E* or *Z* isomers [64,67,68]. However, the *differentia specifica*, which places hydrazides in a category different from that of amides, is the already mentioned presence of a directly attached, subsequent N^β^ atom. It is appropriate for *E*-iminols to be prevalent in amides, while the thermodynamic stability of hydrazides depends on additional factors, including the number and steric expansion of the β nitrogen atom, which prevents a universal isomer from being clearly indicated. The presence of the N^β^ atom determines the possibility of hyper-couplings in hydrazide molecules due to the additional electron pair that adds to the existing electron density [69]. Moreover, in the case of at least monosubstituted hydrazides, it is perfectly distinguishable on a simple infrared spectrum, where these compounds present an additional intense signal shifted by approximately 100–150 cm^−1^ towards shorter waves—compared with their amide analogs with a -CH <group in place of the N atom [70].

Strong signals in the range of 2800–2700 cm^−1^, the so-called Bohlmann bands [71], named after their discoverer, are observed in response to the presence of a substituent of the amine nitrogen atom, in the form of a *sp^2^* carbon atom (which is a ring member) or *sp^3^*. Originally, the study of these bands was used to clarify the stereochemistry of alkaloids, especially those containing the quinolizidine system [72,73]—found in the Egyptian water lily, called the tiger lotus, in the Scotch broom (*Cytisus*, Fabaceae) or in the Andean lupin (*Lupinus*, Fabaceae), such as lupinine or sparteine and its oxo-derivatives. In order to clearly illustrate the core of the Bohlmann effect, the commonly known anomeric effect should be recalled. In the classic approach, there is a preference for the electronegative substituent—attached to the atom adjacent to the heteroatom (most often, the oxygen atom), which is a member of the cyclohexane ring—to assume the axial orientation, despite the fact that if only steric aspects were considered, the equatorial orientation would present much less of a spatial hindrance. The anomeric effect may be regarded as a special type of the Bohlmann effect [73,74,75].

**Figure 6:**
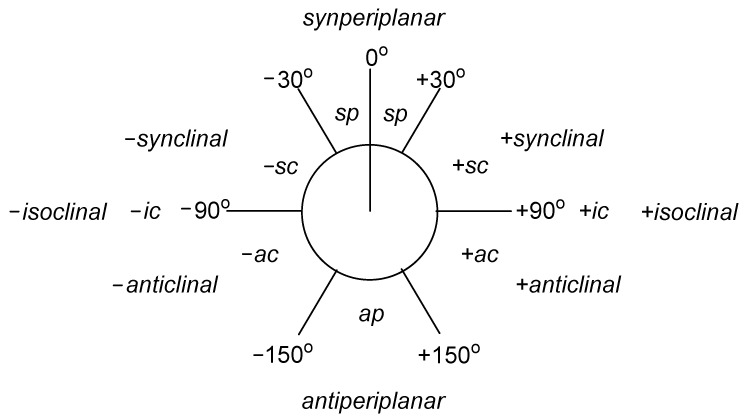
Nomenclature in Newman’s projection depending on the value of the torsion angle [76].

The generalized Bohlmann effect applies to the electron density transfer of lone pairs/lone pair of electrons of an oxygen atom or a nitrogen atom (their participation in the ring is not necessary), the acceptor of which is an anti-bonding CH σ* or CC σ* orbital, oriented antiperiplanarly (Figure 6) towards the orbital on which the lone electron pair is located. The importance of the Bohlmann effect in the context of IR spectroscopy may be seen in the case of amines rather than ethers or alcohols, due to the crucial, lower electronegativity of the nitrogen atom (E = 3.04) in comparison with the oxygen atom (E = 3.44). Electronegativity is defined as the ability of atoms of a given element to attract electrons, and undeniably, lone electron pairs of an oxygen atom are more strongly attracted than the lone electron pair of a nitrogen atom—so its relocation is easier to achieve and results in a greater shift in the infrared spectrum. Moreover, the stronger electronegativity of the elements—the aforementioned oxygen, or fluorine (E = 3.98)—causes a shift of the absorption bands towards higher frequencies, which for ethers or alcohols results in almost complete masking of it. This effect is competitive to the Bohlmann effect and is significantly greater than in the case of nitrogen. It should be mentioned that the antiperiplanarity towards the acceptor orbital for alcohols does not apply to the lone pair on the oxygen atom, but rather to the O-H bond in the hydroxyl group [70].

If we take into account the most energetically stable conformers: antiperiplanar and gauche, then the lowest energetic conformation in at least a monosubstituted amino group means the axial arrangement of both the lone electron pair of the nitrogen atom and at least one C-H or C-C bond. The axial arrangement of the consecutive C-H or C-C bond of a disubstituted amino group (or a ring-closed nitrogen atom) increases the intensity of the Bohlmann bands, and sometimes also causes the appearance of an additional signal in the characteristic range and consequent shifting of the first signal towards waves of lower frequency. The contribution of the lone electron pair of the nitrogen atom to the Bohlmann effect is evidenced by the fact that the introduction of an additional substituent or the formation of an ammonium salt in the IR spectrum is visible as the disappearance of Bohlmann bands. The gauche conformation characterized by the presence of C-H stretching vibrations (not weakened as in the case of the axial position of substituent bonds) is not detectable in the Bohlmann bands region [70].

**Figure 7:**
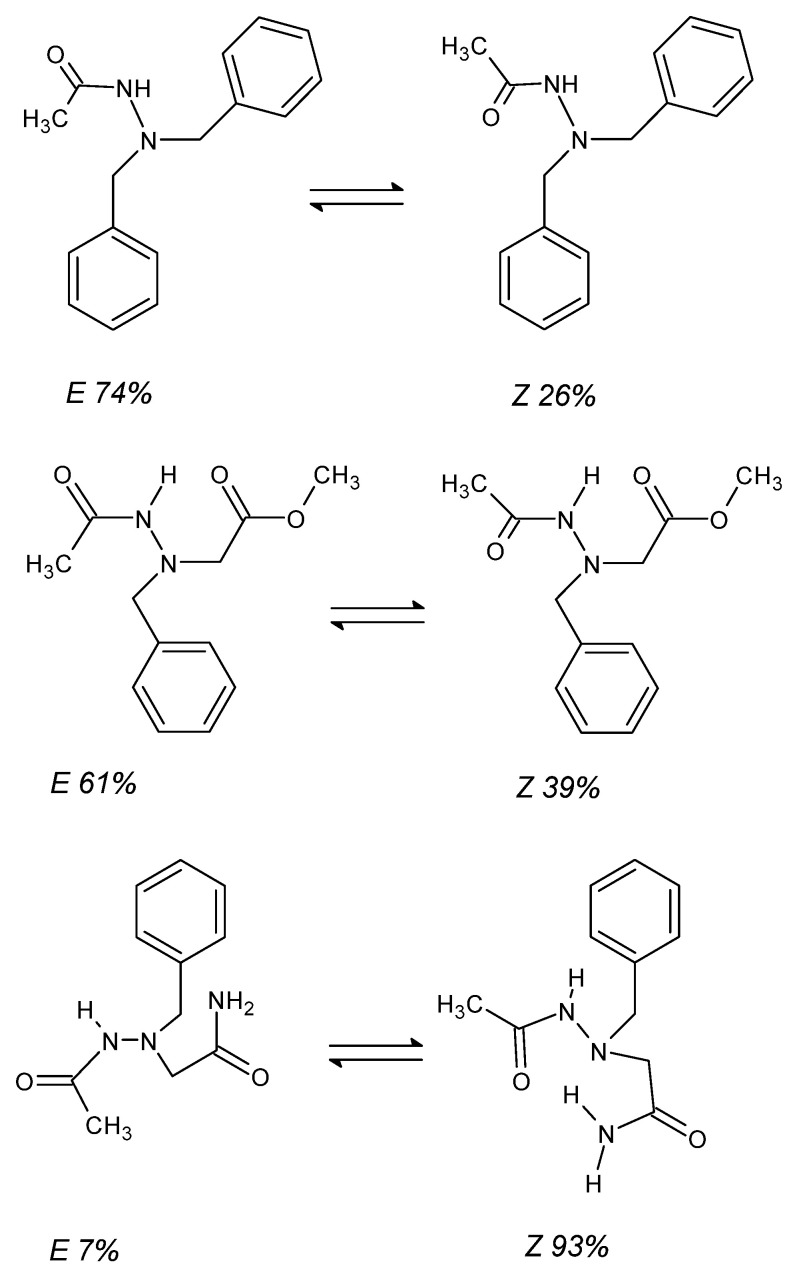
Examples of isomeric equilibria of *E* (**left**) and *Z* (**right**) hydrazides. The *Z* isomer dominates when the folding of the molecule allows for the formation of multiple hydrogen bonds [64].

The topic of hydrazide isomerism must be supplemented with *E*/*Z* descriptors of CN/C=N bond [64,77]. Typically, hydrazides exist in an equilibrium between both isomeric forms (Figure 7), and its shift towards the formation of one of them depends on three overarching factors: the size of the substituents around the bond, the electrostatic repulsion of lone pairs on the oxygen and nitrogen atoms, and the possibility of hydrogen bonding in the folding macromolecules. On the ^13^C NMR spectrum, the isomers are easily recognizable on the basis of the value of the chemical shift δ of the carbonyl signal of the carbon atom, which for *Z* isomers does not exceed 170 ppm, while for *E* isomers, it reaches about 175 ppm. The percentage of isomers is easily calculated from the ^1^H NMR spectrum using the signal integrity of the -NH*_Z_*- and -NH*_E_*- groups, the former of which is shifted more strongly towards higher frequencies [64].

It is worth mentioning that the lone electron pair of the nitrogen atom of the primary amine group gives it the character of a Lewis base, making it able to participate in the formation of hydrogen bonds, intramolecular or with molecules of a polar, competing solvent, and that the iminol form promotes the formation of intramolecular hydrogen bonds, especially in the presence of appropriately non-polar solvents.

### 2.2. Importance

**Figure 8:**
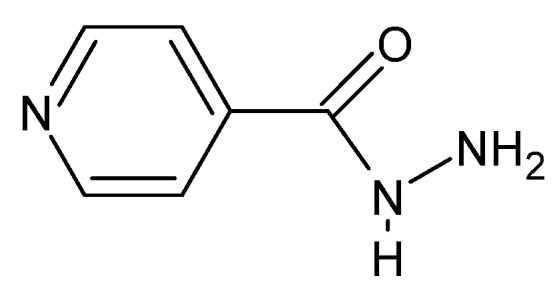
Isonicotinic acid hydrazide (isonidazid).

The documented biological activity of hydrazides confirms their functional affiliation to Schiff’s bases. Hydrazides exhibit broadly understood biocidal properties, including bactericidal properties. *M. tuberculosis* has received special attention since the 1950s. The effectiveness of the synthesized compounds against this bacterium is often compared with the isonidazid (Figure 8), pyridine-4-carboxylic acid hydrazide, which was the first to be studied regarding these properties. In addition, hydrazides also have virucidal (atazanavir is a popular antiretroviral agent used in HIV-1 infections, [65,78,79]), fungicidal and protozoicidal properties.

**Figure 9:**
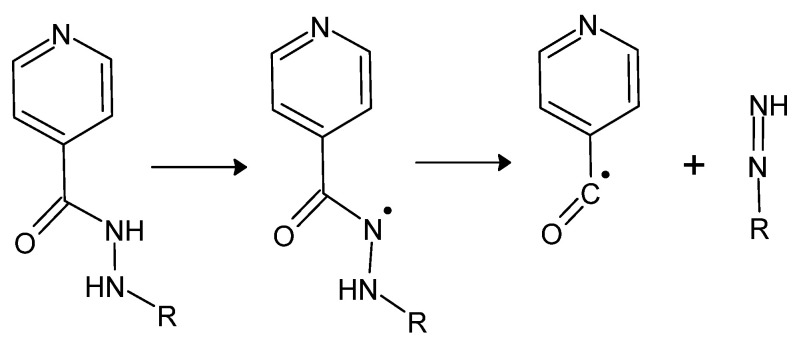
Enzymatic activation of isonidazid or its derivative (*where R is H, an alkyl or aryl substituent*), resulting in the formation of a hydrazyl and ultimately acyl radical and a diazene derivative [80].

The mechanism of action of isonidazid is not fully understood. Most likely, it becomes activated by CP-KatG—catalase-peroxidase—an enzyme inherent in some strains of bacteria, including *M. tuberculosis*, to the form of its acyl radical (Figure 9).

The pyridine-4-carbonyl radical is then coupled with NADH or NAD^+^, and the resulting adduct inhibits the action of InhA reductase (responsible for transferring enoyl and acyl groups). Thus, it hinders the synthesis of mycolic acids—α-branched and β-hydroxylated fatty acids, building bacterial cell walls—contributing to their decomposition and, consequently, also to the death of the bacterial cell [80,81].

**Figure 10:**
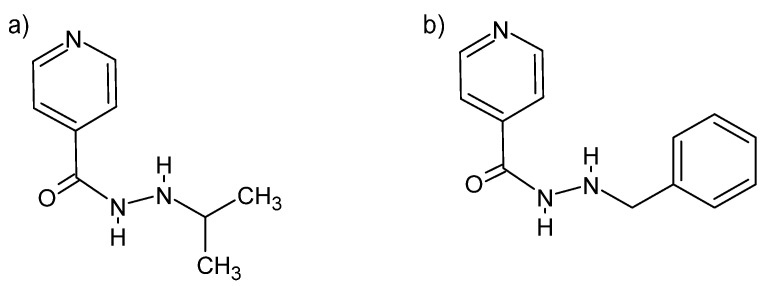
Isonidazid derivatives: (**a**) *N*′-(propan-2-yl)-4-pyridinecarboxylic acid hydrazide and (**b**) *N*′-benzyl-4-pyridine carboxylic acid hydrazide [82].

Some hydrazides can be used to treat depression. Both the *N*′-isopropyl and the *N*′-benzyl isonidazid derivative are of importance as mood modulators (Figure 10). Both act as inhibitors of monoamine oxidase, which causes the deamination of serotonin and norepinephrine [82].

Hydrazides also found chemical application inter alia as catalysts of enantioselective reactions in asymmetric aldol reactions [83], as substrates in regioselective reactions in the preparation of azoheterocycles, such as 1-aryl-1*H*-indazoles [84], or 1,2-disubstituted (in Pd/Cu catalyzed reactions) [85] and multisubstituted alkenes (Mizoroki–Heck reactions) [86] and as a sulfenylating reagents in reactions used to obtain indole thioethers [87]. Hydrazides are also used as protein markers in SSPL (Site-Specific Protein Labeling) methods, in which analyses are performed using spectrometric and electrophoretic (SDS-PAGE) techniques [88] and as modifiers of magnetic beads in the profiling of surface proteins or mapping *N*-glycosylation sites in *A. nige* [89,90]. Primary and secondary hydrazides containing alkene fragments facilitate cyclization in hydroamination and aminocarbonylation reactions [91].

**Figure 11:**
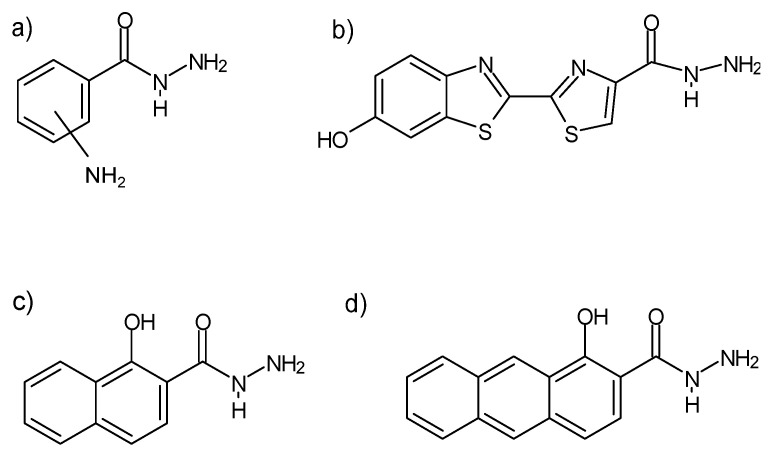
Examples of monosubstituted, hydrazides of (**a**) aminobenzoic acid, (**b**) luciferin, (**c**) 1-hydroksy-2-naphthoic acid, (**d**) 1-hydroxyanthracene-2-carboxylic acid, exhibiting chemiluminescence. The -NH_2_ substituent in the Markush structure (**a**) can assume “ortho” and “meta” positions without losing its chemiluminescent properties [92].

**Figure 12:**
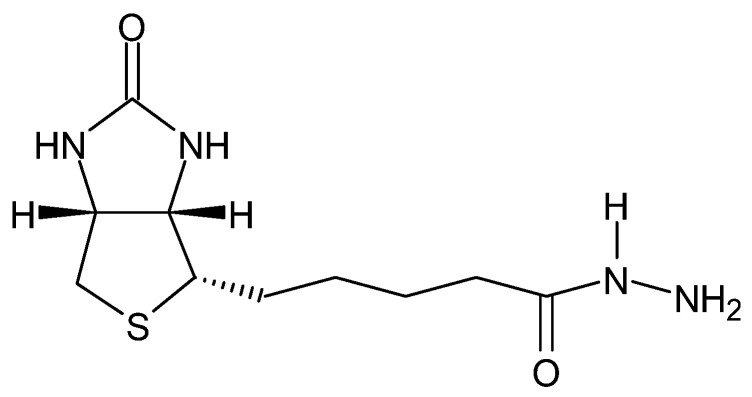
Biotin hydrazide.

Some hydrazides exhibit chemiluminescence (Figure 11) [92], which allows for their use in the labeling of, e.g., chromate(VI) ions without reducing them to Cr(III)—rhodamine B and rhodamine G6 hydrazides [93], or deoxyribonucleic acid (Figure 12)—biotin hydrazide [94].

As aza-peptides, they find use as inhibitors of serine and cysteine proteases due to their lower susceptibility to enzymatic hydrolysis in comparison to their peptide analogs and greater metabolic stability [95,96]. Moreover, the appropriate inclusion of an aza-amino acid in the synthetic peptide chain stabilizes its β-turns [97].

**Figure 13:**
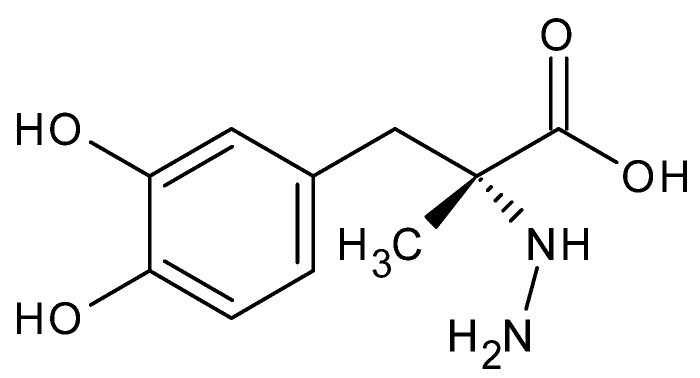
Carbidopa-*N*-amino-α-methyl-3-hydroxy-L-tyrosine.

A useful example of a hydrazine derivative is carbidopa hydrazine acid (Figure 13), co-administered with the dopamine precursor (*S*) 3,4-dihydroxy-L-phenylalanine (levodopa), in the treatment of Parkinson’s disease. This hydrazine acid regulates the rate of dopamine release, prolonging the effect of the drugs [82].

**Figure 14:**
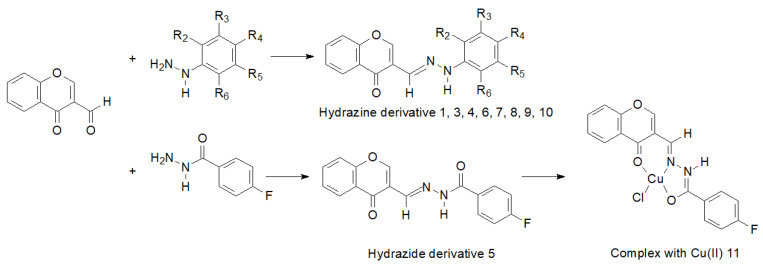
Synthesis of hydrazine and hydrazide derivatives of 3-formylchromone. Hydrazine derivatives of 3-formylchromone: **1**: R2=R5=F, R3=R4=R6=H; **3**: R4=CF3, R2=R3=R5=R6=H; **4**: R3=R5=CF3, R2=R4=R6=H; **6**: R2=R3=R5=R6=F, R4=H; **7**: R2=CH3, R5=F, R3=R4=R6=H; **8**: R2=R4=R6=F, R3=R5=H; **9**: R2=R3=R4=R5=R6= F, **10**: R2=CF3, R3=R4=R5=R6=H.

Interesting results of research on the synthesis, structure and biological activity of hydrazine and hydrazide derivatives of 3-formylchromone were presented by Słomiak et al. [98]. They synthesized a number of hydrazine derivatives and a hydrazide derivative which they complexed with Cu (II) (Figure 14).

They found that concentrations of 0.01–1250 μmol/L influenced cell proliferation. In the case of two cell lines: the L929 cell line (mouse fibroblast cell line) and the EA.hy926 cell line (human umbilical cord vein, somatic cell hybrid), these compounds demonstrated antiproliferative activity and stimulation of proliferation.

### 2.3. Classification

**Figure 15:**
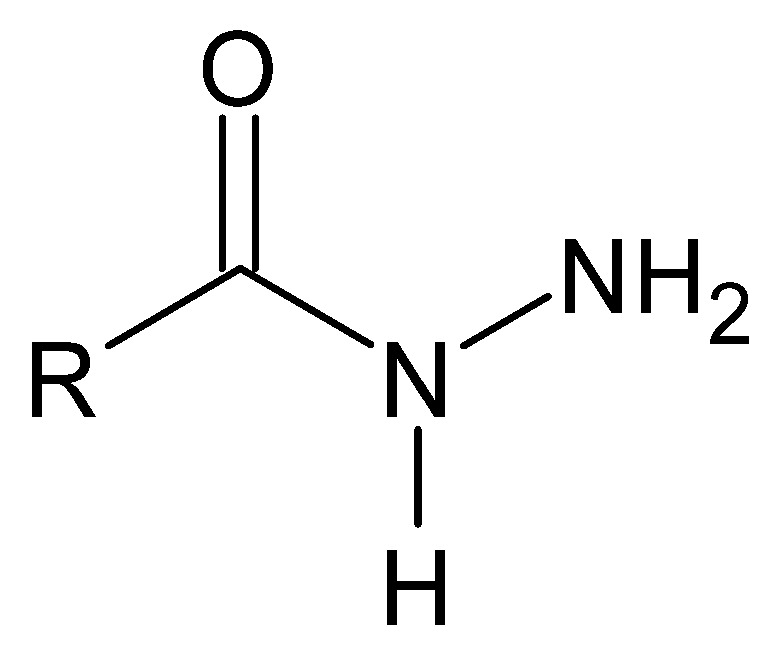
Structure characteristic for carbonyl hydrazides, where R is an alkyl or aryl substituent.

The simplest criterion for the classification of unsubstituted hydrazides, i.e., those containing a free amino group, is to consider the type of atom connected directly to the nitrogen atom of the secondary amino group. The most commonly studied are carbonyl-type hydrazides (Figure 15), obtained by the nucleophilic substitution of an ester of a suitable carboxylic acid with hydrazine. The reaction is sometimes referred to as the hydrazinolysis of carboxylic acid esters [99].

The obtainment of the first hydrazides was reported by German scientists as early as 1892, as a result of the reaction of fatty acid esters with hydrazine (G. Schöfer) and the reaction of glycolic acid ester with hydrazine (N. Schwan), and more in 1895 by the aforementioned authors and T. Curtius, including the simplest hydrazide, i.e., formic acid hydrazide [100]. In 1968, R. Slagel obtained them through a less conventional method, in addition to the carboxylic acid ester, using a disubstituted, unsymmetrical hydrazine and an epoxide [101].

**Figure 16:**
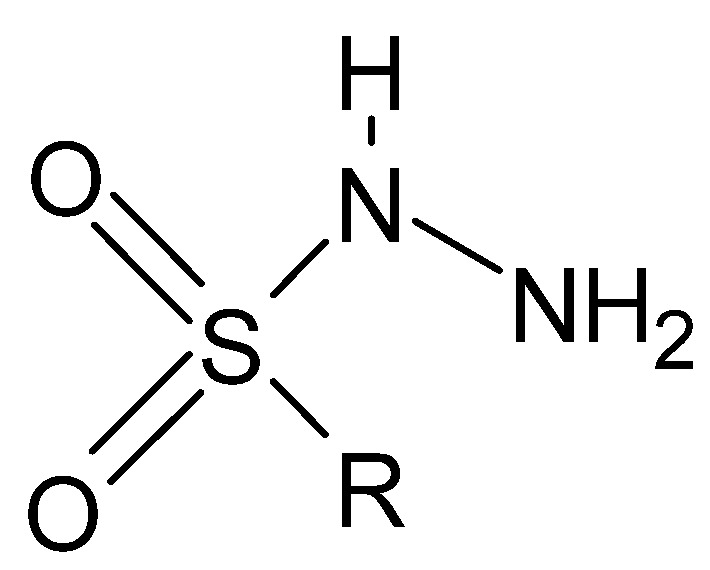
Basic structure of sulfonyl hydrazides, where R is an aryl group.

Of lesser interest are sulfonyl hydrazides (Figure 16), in which the connection with the R group is most often formed through a sulfonyl group, with the sulfur atom bonded directly to the aromatic ring.

**Figure 17:**
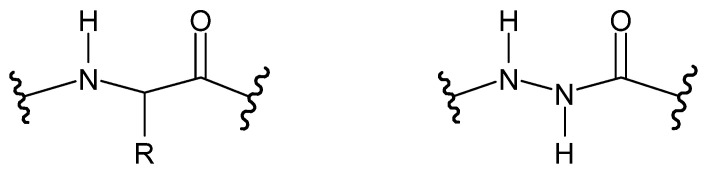
Peptide (**left**) and aza-peptide (**right**), where R is a side chain.

**Figure 18:**
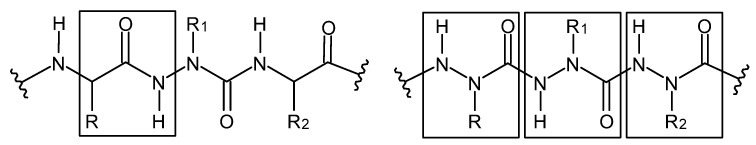
Aza-peptide (**left**) and azatide (**right**), where R, R_1_ and R_2_ are side chains—hydrazide bonds are framed.

However, the simplest criterion is not the only one. Some hydrazides can be called aza-amino acids, that is, amino acids enriched with an additional amino group, simultaneously directly connected to the amino N atom and carbonyl C atom, that replace the α-CH moiety. If said aza-amino acid is a part of a poly-chain of at least 10 members, it forms the aza-peptide (Figure 17 and Figure 18) [95].

A special type of aza-peptide, called an azatide, is a biopolymer made exclusively of α-aza-amino acids [102].

**Figure 19:**
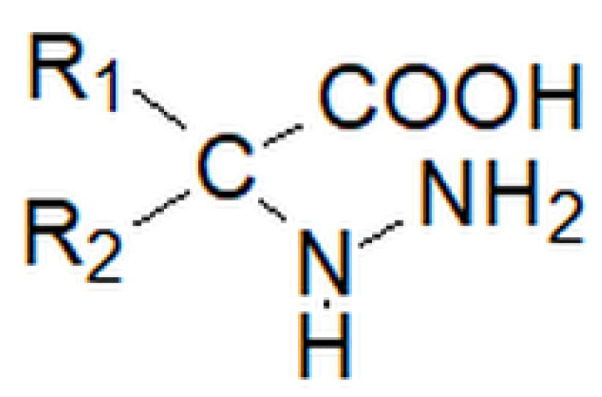
Structure of hydrazine acid, where R_1_ and R_2_ may be a hydrogen atom, an alkyl group or an aryl group.

Hydrazine acids (Figure 19) are derivatives of amino acids in which the amino group -NH_2_ is replaced with the hydrazine group -NH-NH_2_ [103].

It is worth mentioning that due to the possibility of folding and the formation of hydrogen bonds between non-adjacent hydrogen atom acceptors and hydrogen atoms derived from -OH and -NH- groups, the share of the *E* isomer in the isomeric equilibrium decreases with the elongation of the chain [64,104]. Additionally, in the case of participation in the hydrogen bond of the H atom coming from the primary amine group, two singlets (of a significant chemical shift in relation to each other, e.g., δ 2.60 ppm) may appear in the ^1^H NMR spectrum, one on each side of the -NH*_Z_*- signal [64]. Participation in the hydrogen bond causes the hydrogen atoms of the -NH_2_ group to appear in the spectrum as separate signals.

One cannot fail to mention the interesting combinations called metallohydrazides: precursors of hydrazides, which can also be considered their isolobal analogues. The concept of isolobality introduced by Roald Hoffmann is based on the theory of frontier (border) orbitals HOMO and LUMO by Kenichi Fukui, assuming that, in simplified terms, the reactivity of a molecule or its fragment results from the properties of its frontier orbitals, i.e., valence active orbitals. It describes similarity in the number, symmetry, approximate energy and shape of orbitals and in the number of their electrons between organometallic compounds and known organic ligands, helping to determine the electronic structure and, consequently, the reactivity of the former [105,106,107].

**Figure 20:**
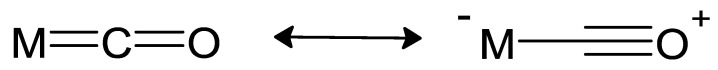
Resonance forms of the M–C–O bond [55].

Essential is the presence of an even-electron Lewis base as the electron donor in the ligand [106]. Naturally, in the organometallic complex the transition metal atom also participates —in the described complexes, ones belonging in the 6th–8th groups in the periodic table. Tetra- and pentacarbonyl hydrazinecarbonyl complexes (Fisher carbene type) lead to the production of corresponding hydrazides due to the isolobality of the metal = carbon double bond as regards the double bond of the carbonyl group in the hydrazide even as a result of mild oxidation. The complexation of a metal atom with carbonyl ligands is twofold in nature, i.e., simultaneously of a coordinate σ-bond and a π-type return (redonor) bond. The first and most important of them arises as a result of overlapping of the empty hybridized orbitals (formed from the *s*, *d* and *p* orbitals) on the metal atom and the filled hybridized HOMO orbital on the carbon atom in the carbon monoxide(II) molecule with the donation of a lone electron pair by the latter. The second, on the other hand, is the consequence of overlapping of the filled *d*π orbitals or hybridized *dp*π orbitals on the metal atom and the unfilled LUMO orbital on the carbon monoxide(II) carbon atom after the initial transfer of electrons from the filled π orbital of the CO molecule to the unfilled *d*-orbital of the CO molecule and their subsequent redonation to the available π* orbital on the carbon atom. Thus, a resonance hybrid is formed (Figure 20), in which the multiplicity of the bond between the metal and carbon atoms is between 1 and 2, and the bond between the carbon and oxygen atoms—between 2 and 3 [8,107,108,109,110].

Transition metal carbonyls easily give Fisher-type carbenes with organolithium compounds, and in a two-step substitution reaction at a carbon atom with *sp^2^* hybridization (that consists of a nucleophilic addition followed by elimination) with an amine (in this case, hydrazine), leading through a tetrahedral intermediate—hydrazinecarbenes [110]. These compounds can be used in RCM (Ring-Closing Metathesis) reactions [111] and in reactions with peptide nucleic acid monomers—DNA and RNA mimetics, in which the main phosphate-sugar chain is replaced with a polyamide chain composed of *N*-(2-aminoethyl)-glycine units [112]—in order to modify their properties, including increasing their lipophilicity and thus, the ability to penetrate cell membranes, which may increase their use in molecular biology and medicine [113].

**Figure 21:**
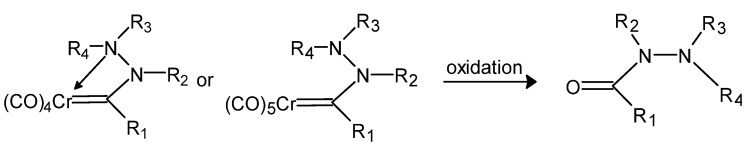
Tetra- and pentacarbonyl hydrazinecarbonyl complexes and their oxidation product carbonyl hydrazid (*R_1_*, *R_2_*, *R_3_ and R_4_ are an alkyl group*) [107].

The reaction to obtain hydrazides should not be carried out without considering the sensitivity of the expected products to further oxidation (Figure 21). Molecular iodine obtained in situ can be a safe, mild oxidant. The reagent kit used for this purpose is an iodide anion oxidized with sodium borate in a neutral pH water–ethyl acetate buffer with the addition of potassium bicarbonate, which results in the gradual release of molecular iodine into the reaction mixture [107].

## 3. Dihydrazides

### 3.1. Structure

**Figure 22:**
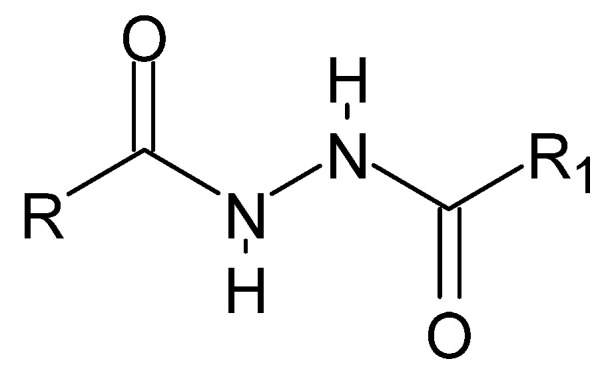
The basic structure of dihydrazides, where R and R_1_ is an alkyl, aryl, or hydrogen group.

The plurality of dihydrazide compounds (Figure 22), similar to that of hydrazide–hydrazones, results from the variety of alkyl or aryl fragments and their substituents that can be linked by a diamide bridge. The symmetrical structure determines the nomenclature: each time, we start naming with the carbonyl group, invariably obtaining a monosubstituted hydrazide. Thus, including both reading possibilities, the name mentioned above can be adopted.

The synthesis of dihydrazides—methanoic and ethanoic acid dihydrazides—was first described by T. Curtis, N. Schwann and G. Schöfer in 1895 [100].

### 3.2. Importance

Dihydrazides are important as antibacterial, antifungal and antiparasitic agents. The fact that it is the hydrazide moiety, which is the key to the biological activity of similar compounds, was already reported in the research by Raymond Cavier and Richard Rips in 1965, in which they observed that the replacement of the diisopropylidenemalonyl hydrazide moiety with an amide moiety reduces the compound’s activity against the nematode *S. obvelata* [114].

An example of another property of some dihydrazides is chemiluminescence, i.e., the emission of radiation manifested by the light effect (ultraviolet, visible light and infrared), which results from the recombination of the electron–hole pair in an electronically excited product or an intermediate product, resulting from the action of an external factor, which in this case is a chemical reaction, on a given compound [115]. One of the earliest described chemiluminophores—as early as 1928 [116], commonly called luminol, is 3-aminophthalic acid hydrazide, with the capacity to emit light at λ = 425 nm (blue light) [92]. The exchange of the carbonyl groups with the secondary amino groups while maintaining the symmetry (amide groups connected through the carbon atoms of carbonyl groups) causes the compounds to lose their chemiluminescent properties. They are also negatively affected by the presence of electron-withdrawing substituents (-Cl, -NO_2_), while electron-donating substituents (-NH_2_, -OH) strengthen them [92]. The chemiluminescent properties allow the use of dihydrazides, as they are highly sensitive to pH changes and are indicators of the endpoint of acid–base titration (*N*′-formyl-rhodamine B dihydrazide) [117], as DNA probes and in immunoassays (luminol) [118].

**Figure 23:**
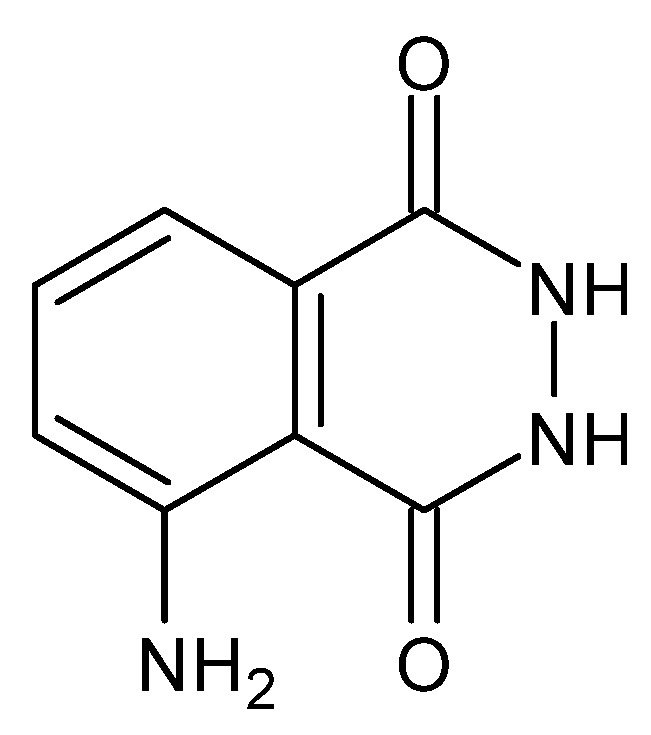
Structure of 3-aminophthalic acid hydrazide (luminol) [92].

**Figure 24:**
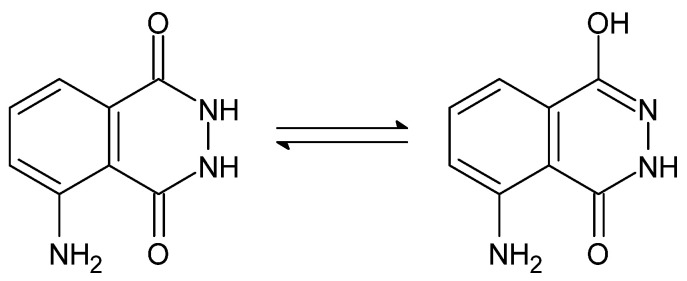
Amide-iminol tautomerism of luminol [92].

The presence of the amide-analogous -C(=O)-NH-NH- group enables the occurrence of the aforementioned amide-iminol tautomerism, presented below (Figure 23 and Figure 24) [92].

Dihydrazides are also used as catalysts in highly enantioselective reactions leading to the formation of asymmetric aldols [83].

## 4. Hydrazones

### 4.1. Structure

**Figure 25:**
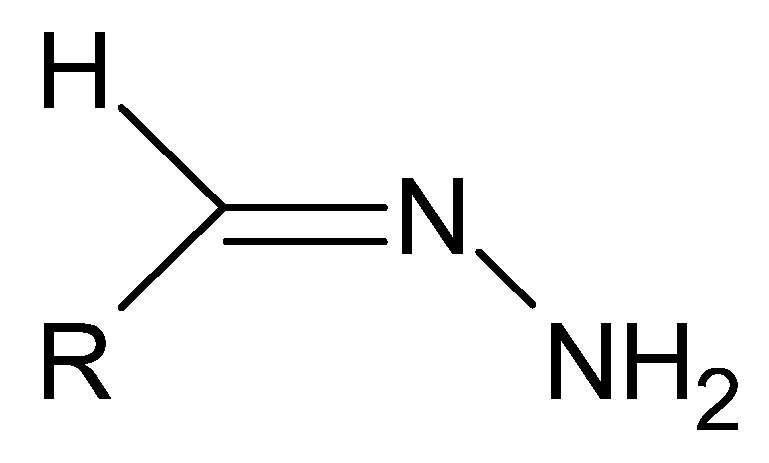
The basic structure of hydrazones, where R is an alkyl or aryl group.

**Figure 26:**
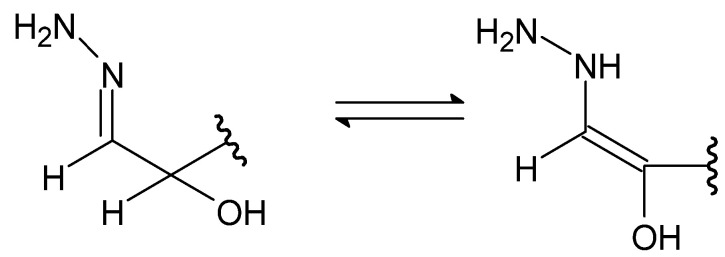
The imine–enamine tautomerization of hydrazones.

A special type of hydrazides are hydrazones, the main distinguishing feature of which is the presence of an imine bond in their moiety (Figure 25), which in turn participates in imine–enamine tautomerization (Figure 26) due to the presence of the α-hydrogen atom.

Characteristic signals are visible in the ^1^H NMR spectrum in the form of a singlet with a chemical shift of δ 8.16–8.67 ppm for -CH= and a singlet with a chemical shift of δ 10.45 12.25 ppm for -NH- [119].

The N-N bond can be reduced to the -NH_2_ group [120] or the whole hydrazone molecule can be reduced to a hydrazide by reductive acylation [121]. The C=N bond is susceptible to a nucleophilic attack. It can be hydrolyzed, oxidized or reduced—it willingly restores the C=O carbonyl group. It can undergo the nucleophilic addition of an organometallic compound (containing Li, Mg, Ce and Yb atoms), as well as of the intramolecular group -SH [107,122]. Under appropriate conditions, hydrazones can react with α,β-unsaturated aldehydes, giving interesting dihydrazide connections with a lactam ring [123].

### 4.2. Importance

Hydrazones have uses, among others, as pesticides, insecticides, nematicides, rodenticides or plant-growth regulators [124].

Sulfonyl hydrazones are important as antidepressants, analgesics, anti-inflammatory, anti-cancer, antifungal, and antibacterial agents—they can act as strong inhibitors of tyrosine phosphatase B (PtpB) of bacteria from the *M. tuberculosis* strain [125] and are antidiabetic [126]. This particular type of hydrazone is often tested for antimicrobial properties alongside its analogues—sulfonamides [125]. Equally common is the use of aryl hydrazones, in which the hydrazone moiety is directly linked to the aromatic ring, in medicine [127].

As with Schiff bases, hydrazones are considered multidentate ligands for their chelating capacity. Complexes of hydrazones with transition metal ions may show increased antimicrobial activity compared to uncomplexed ligands, where it is the metal ion that has the capacity to disturb cellular activity, while the ligand “assists”, increasing the lipophilicity of the ion’s environment, thus allowing for its penetration of the cell membrane [61,128,129,130].

**Figure 27:**
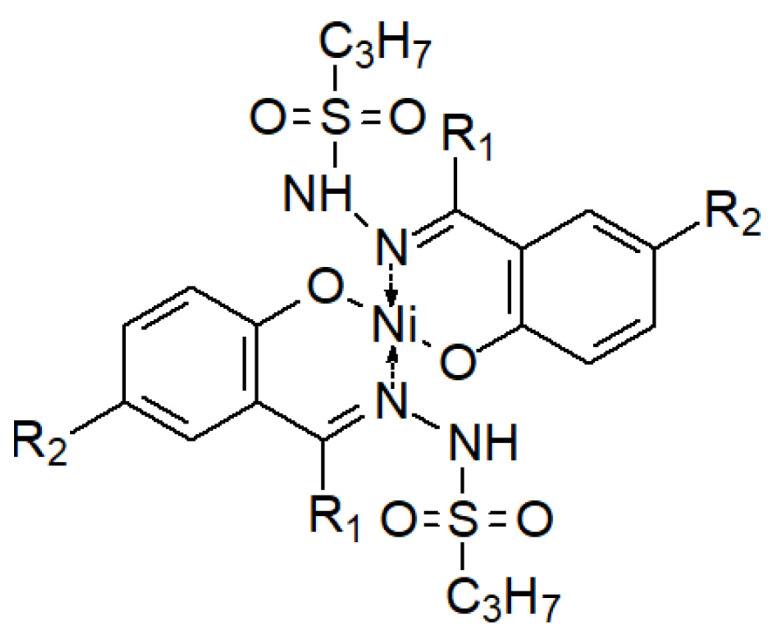
A complex of two hydrazone ligands and one nickel(II) ion [131]. R1 and R2 are substituents as described in Table 1.

Less frequently, the opposite phenomenon occurs—a decrease in the antibacterial activity of the sulfonyl hydrazones and their complexes (Figure 27) in relation to their ligands (Table 1), which is explained by the fact that the electron density is reduced on the donor (O, N) atoms involved in the formation of coordination bonds with the tran-sition metal ion in the complex [131].

### 4.3. Classification

**Figure 28:**
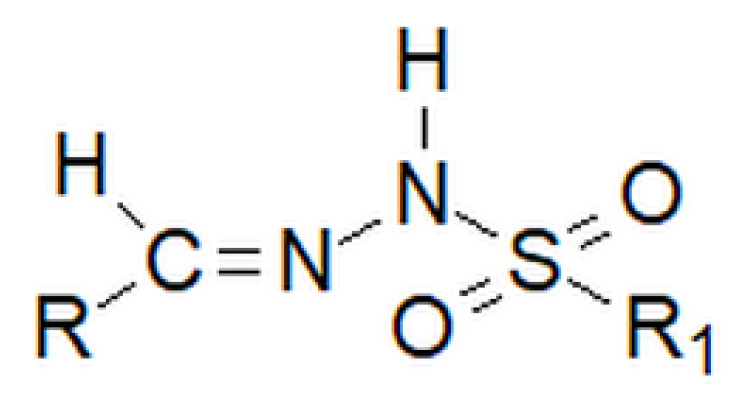
Basic structure of the sulfonyl hydrazone, where R and R_1_ are alkyl or aryl groups.

Among hydrazones, as well as among hydrazides, besides carbonyl, one can distinguish sulfonyl hydrazones. However, the sulfonyl group does not replace the nitrogen–carbon bond inherent to hydrazones, but is attached to the amino group, which is terminal in classic hydrazones (Figure 28 below).

The oxidation of thiol leads to sulfonyl acid, and with chlorinating reagent (SOCl_2_, POCl_3_, PCl_5_ and cyanuric acid chloride)—to sulfonyl acid chloride, which can further react with hydrazine and aldehyde or ketone to give sulfonyl hydrazone [126]. A special type of sulfonyl hydrazone is 4-toluenesulfonylhydrazone, obtained by reacting an aldehyde or ketone with tosylhydrazide, which is the product of the reaction of tosyl chloride and hydrazine [132].

## 5. Hydrazide–Hydrazones

### 5.1. Structure

**Figure 29:**
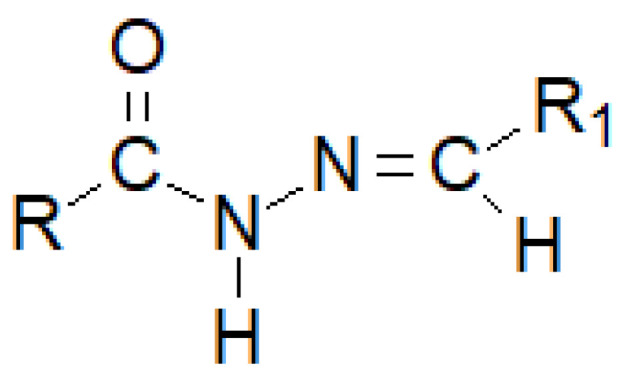
The basic structure of a hydrazide–hydrazone, where R and R_1_ are either alkyl or an aryl groups.

Hydrazide–hydrazones are a numerous group of hybrid molecules which can connect diametrically different alkyl or aryl fragments through an unsymmetrical amide–imine bridge -C(=O)-NH-N=CH-, which builds the heterogeneity of this group of compounds (Figure 29). Its name unsurprisingly comes from the dual possibility of reading the order of atoms in a moiety. If the nomenclature begins with the carbonyl group, we can say that we are dealing with monosubstituted carbonyl hydrazides, while if we regard the imine bond as the beginning, the—monosubstituted—hydrazone clearly appears in front of us.

The hydrazide–hydrazone moiety can be identified via spectroscopic methods. The IR spectrum shows signals at around 3050 cm^−1^, 1650 cm^−1^ and 1550 cm^−1^, corresponding to the -NH-, C=O and C=N group, respectively. Two singlets appear in the ^1^H NMR spectrum, one in the range of δ 8–9 ppm and the other in the range of δ 10–13 ppm, signals corresponding to the -CH= and -NH- group, respectively. In the ^13^C NMR spectrum, there are signals of carbon atoms from the CH= and C=O group in the range of δ 145–160 ppm and δ 160–170 ppm, respectively [133].

### 5.2. Importance

**Figure 30:**
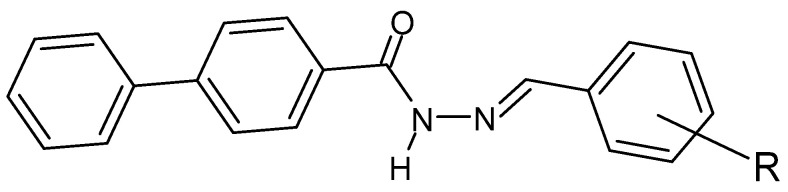
Hydrazide–hydrazone derivative of biphenyl-4-carboxylic acid, where R = NO_2_, -Cl or -Br [135].

Hydrazide–hydrazones are synthesized in search of effective antibacterial and antifungal agents—especially due to the growing problem of antibiotic resistance [134]. In many cases, it is the presence of electron-withdrawing aromatic ring substituents that is essential for the antimicrobial activity of hydrazide–hydrazones. Studies on the biological activity of, among others, the following compound (Figure 30) provide an example of the mentioned relationship. The lowest MIC values (μg/mL) are shown for the chlorine substituent in position 2, against *E. coli*, *S. aureus* and *B. subtilis*, 0.31, 0.62 and 0.31, respectively. They were compared with the MIC values of ciprofloxacin (an organic antimicrobial compound inhibiting bacterial DNA topoisomerase) of 0.01, 0.15 and 0.12, respectively. On the other hand, the highest efficacy among the tested derivatives against *C. albicans* was recorded for the -NO_2_ substituent in position 2 (MIC = 0.31 μg/mL). The value was compared with clotrimazole (an organic compound with antifungal activity that inhibits the biosynthesis of sterols that build fungal cell membranes), for which the MIC value is 0.10 μg/mL [135].

**Figure 31:**
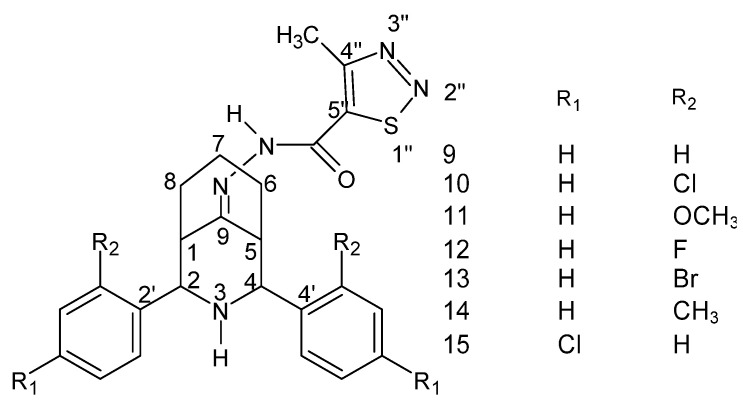
Structure of the 2r, 4c-diaryl-3-azabicyclo [3.3.1] nonan-9-one-4-methyl-1,2,3-thiadiazole-5-carbonyl hydrazide–hydrazone [136].

The following compounds (Figure 31) were tested against streptomycin and against *B. subtilis*, *K. pneumoniae* and *E. coli* and showed lower MIC values for the chloro, fluoro and *para* substituents (Table 2). Moreover, lower values were also recorded for the above-mentioned derivatives and for the derivative with the chlorine atom in the “*ortho*” position (Table 3) against fluconazole and against *A. flavus*, *A. niger*, *C. albicans* and *Candida6* [136].

**Figure 32:**
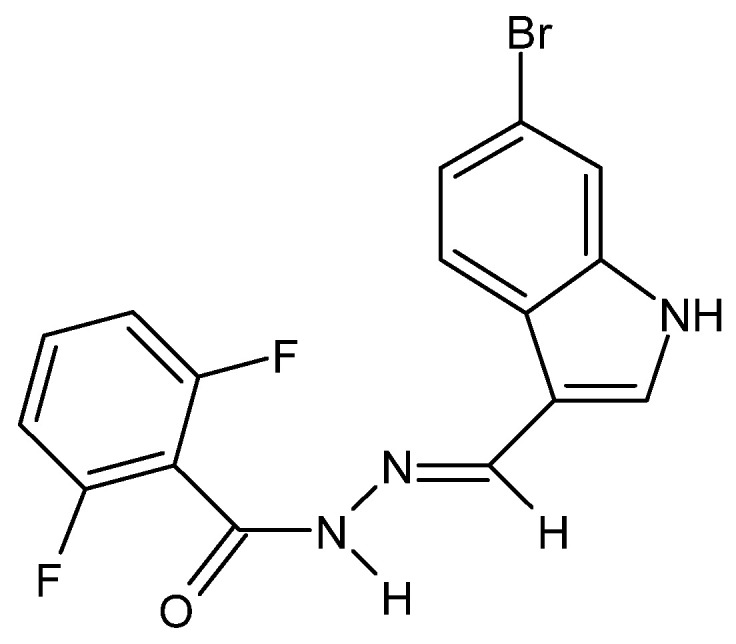
Derivative of 2,6-difluorobenzoic acid and 6-bromoindole—hydrazide–hydrazone with anti-cancer properties [77].

There have also been reports in the literature on the anti-cancer properties of hydrazide–hydrazones, e.g., in the case of colorectal cancer acting by increasing the permeability of the outer mitochondrial membranes of neoplastic cells. The cytotoxic activity of the hydrazide–hydrazone derivative of 2,6-difluorobenzoic acid and 6-bromoindole (Figure 32) was confirmed against the HTC-116, DLD-1 and SW-620 cell lines, while its lack—against healthy fibroblasts of the L929 cell line [77].

For the HTC-116 cell line, the pathway of programmed cell death has been established, starting with the inhibition of Bcl2 proteins, which regulate this process and act in an anti-apoptotic manner, and, consequently, regulate the release of pro-apoptotic cytochrome c. Caspases-9 and -3, which regulate the production of reactive oxygen species, take over further control of the process [77]. Cytochrome c binds Apaf-1, which is the first factor activating the apoptotic protease-activating factor 1. The formation of this complex (apoptosome) leads to the activation of procaspase-9 to the initiator caspase-9, which in turn activates the executive caspases, caspase-3 and caspase-7, of which caspase-3 is the master caspase and caspase-7 is the helper caspase. Caspase-9 begins the secretion of reactive oxygen species that have a proteolytic effect on the cell subjected to apoptosis, and caspase-3 acts as an inhibitor, ending the process [137].

Hydrazide–hydrazones are also used as antidiabetic agents in type II diabetes [138,139]. They play the role of non-competitive antagonists of the glucagon receptor—thus, they inhibit the glucagon-induced processes: glycogenolysis and gluconeogenesis, which in turn leads to a reduction in blood sugar levels.

**Figure 33:**
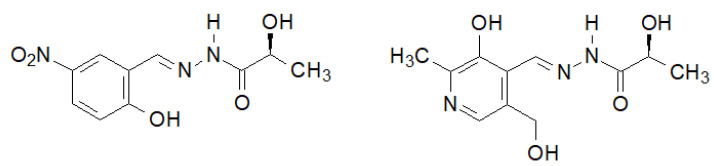
New hydrazide–hydrazones of lactic acid with antibacterial activity.

A very interesting biological activity of the hydrazide–hydrazone of lactic acid was described by Noshiranzadeh et al. [140]. They synthesized a number of this type of lactic acid derivatives, two of which (Figure 33) showed particular activity against the selected bacterial strains (Minimum Inhibitory Concentration MIC = 64–128 µg/mL), which turned out to be lower than the reference gentamicin.

**Figure 34:**
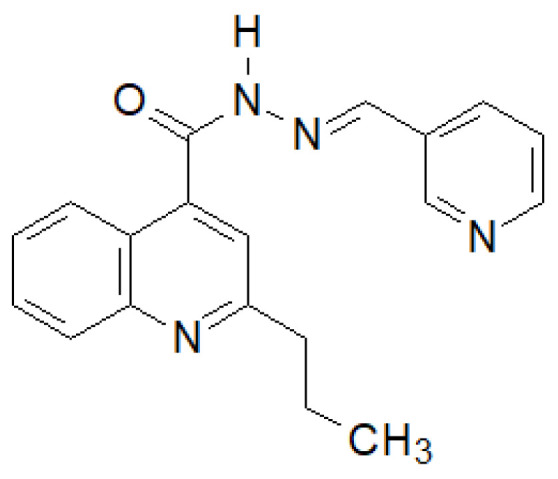
Quinoline derivative with significant antibacterial properties.

Olayinka et al. synthesized several 2-propylquinoline-4-carboxylic acid hydrazide–hydrazones [141]. The compound shown in the Figure 34 showed the highest activity against six strains of bacteria.

The authors showed that the presence of an electron-donating substituent in position 4 and an electron-withdrawing substituent in position 2 is of key importance for the activity of the derivatives obtained.

**Figure 35:**
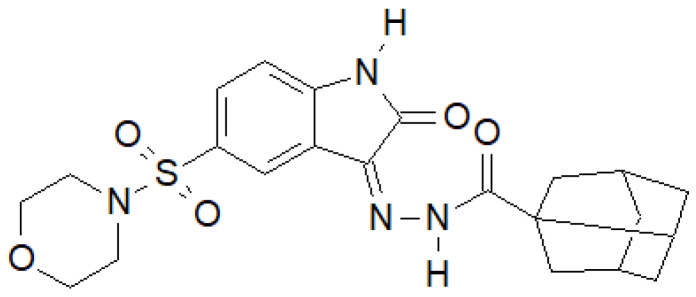
Indol-2-one derivative with antibacterial activity.

Indole-2-one was used by Salem et al. to obtain a series of hydrazide–hydrazones, of which the compound in Figure 35 had the most interesting properties [142].

For the above compound, they performed an activity assay against DNA gyrase isolated from *S. aureus*, which showed that its half-maximal inhibitory concentration was IC_50_ = 19.32 ± 0.99 µM, while for the reference ciprofloxacin, IC_50_ = 26.43 ± 0.64 µM.

**Figure 36:**
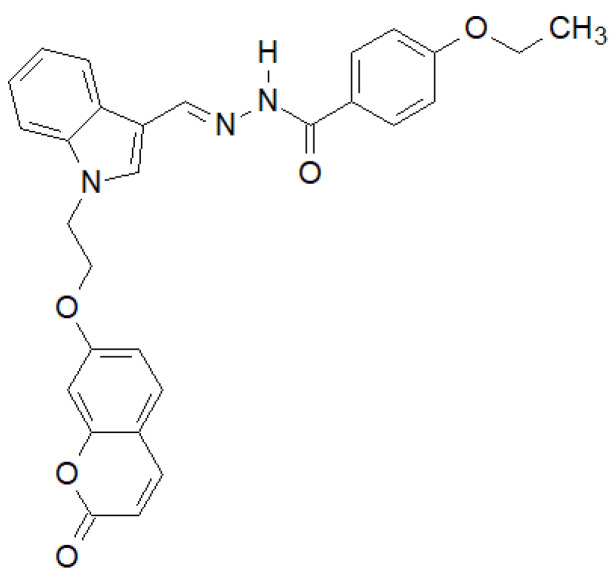
*N*-substituted indole derivative with antibacterial properties.

Of the *N*-substituted indole derivatives synthesized by Tiwari et al., the compound shown in Figure 36 showed the highest activity against Gram-positive bacteria [143]. In the case of *E. coli* MTCC 433 and *B. subtilis* MTCC 1427, it showed higher activity than the reference Chloramphenicol.

**Figure 37:**
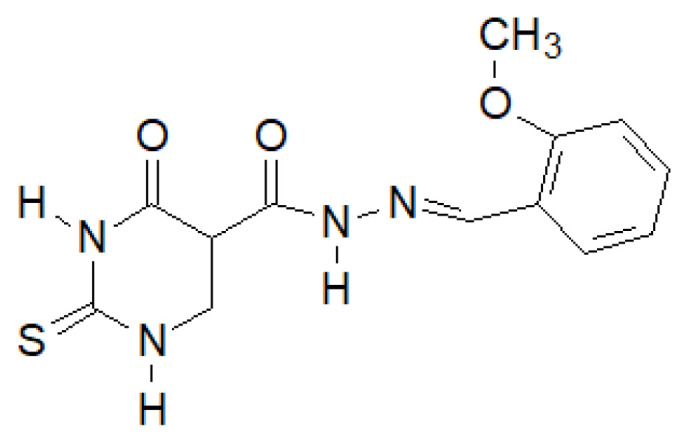
*N*-(2-Thiouracil-5-oyl)hydrazone derivative with antibacterial activity.

El-Etrawa et al. prepared a number of 2-thiouracil derivatives, of which the following compound (Figure 37) turned out to be the most active against *E. coli*, *P. aeruginosa* and *S. aureus* [144].

**Figure 38:**
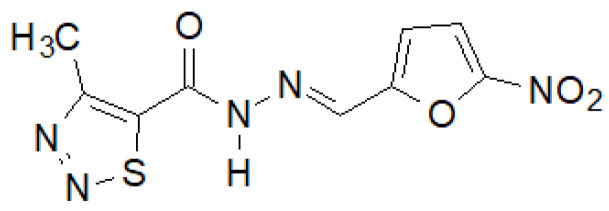
4-Methyl-1,2,3-thiadiazole-carboxylic acid hydrazide derivative active against a panel of bacterial strains.

Recently, Paruch et al. synthesized 1,2,3-thiadiazole hydrazide–hydrazones, of which the compound shown in Figure 38 showed the highest activity [145].

This compound showed activity against almost all tested bacterial strains, and in the case of *S. epidermidis* ATCC 12228 and *M. luteus* ATCC 10240, even two and eight times higher, respectively, than the reference nitrofurantoin.

**Figure 39:**
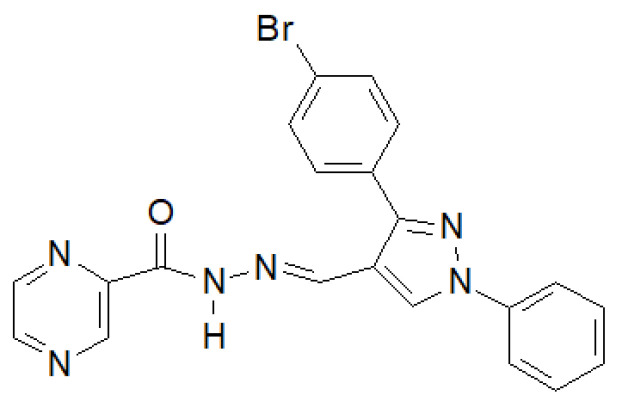
Pyrazine derivative with antitubercular properties.

In 2020, the results of the synthesis and biological tests of eugenol hydrazide–hydrazones were published [146]. The compound in the Figure 39 showed the highest activity against *M. tuberculosis* H37Rv.

## 6. Conclusions

The group of compounds presented in this article seems to be exceptional due to the already known and predicted biological activity. New groups of organic compounds are still being described, the combinations of which may form a group of extremely desirable compounds with antibiotic properties, suitable for an attempt to challenge the problem of antimicrobial resistance. In the instance of the results described in the article by R. C. N. Reis et al. from 2008 [147], in which the activity of the coupling products of D-ribonic acid hydrazide and long-chain aldehydes and the hydrazinolysis products of D-ribo-1,4-lactone with *N*,*N*-disubstituted hydrazines was tested against *M. tuberculosis*, *S. aureus*, *E. coli* and *C. Albicans*, versus i.o. rifampicin, penicillin-G, chloramphenicol and nystatin, the authors noted that the greater the activity of the coupling products against *M. tuberculosis* and *S. aureus*, the longer the attached hydrocarbon chain was. Moreover, the results of examining the biological activity of monosaccharide hydrazide derivatives and their complexes with transition metal ions in relation to selected microbial strains may turn out to be remarkable, and may be used to create a complete description of their significance and enable the comparison of properties with hydrazide derivatives already described in the literature.

## Figures and Tables

**Table 1:** Summary of MIC values (μg/mL) of selected hydrazone derivatives (Figure 27 below) and their complexes with the Ni(II) ion against selected bacterial strains [131].

No.	Type of Substitution; Ligand/Complex	MIC Values (μg/mL)			
		*B. subtilis*	*B. magaterium*	*S. aureus*	*S. enteritidis*
**1**	R_1_=R_2_=H; ligand	266	242	145	242
	R_1_=R_2_=H; complex	595	541	541	595
**2**	R_1_=H, R_2_=CH_3_; ligand	282	256	154	256
	R_1_=H, R_2_=CH_3_; complex	569	683	626	626
**3**	R_1_=CH_3_, R_2_=H; ligand	256	307	256	282
	R_1_=CH_3_, R_2_=H; complex	569	626	569	626
**4**	R_1_=R_2_=CH_3_; ligand	324	297	270	297
	R_1_=R_2_=CH_3_; complex	717	657	657	597

**Table 2:** Summary of MIC values (μg/mL) of selected hydrazide–hydrazone derivatives against selected bacterial strains [136].

No.	Type of Substitution/Reference Compound	MIC Value (μg/mL)		
		*B. subtilis*	*K. pneumoniae*	*E. coli*
1	*para*-Cl	6.25	6.25	12.5
2	*para*-F	6.25	6.25	12.5
3	*para*-Br	6.25	6.25	12.5
4	*streptomycin*	12.5	12.5	25.0

**Table 3:** Summary of MIC values (μg/mL) of selected hydrazide–hydrazone derivatives against selected fungal strains [136].

No.	Type of Substitution/Reference Compound	MIC Value (μg/mL)			
		*A. flavus*	*A. niger*	*C. albicans*	*Candida6*
1	*para*-Cl	12.5	12.5	6.25	6.25
2	*orto*-Cl	12.5	12.5	6.25	6.25
3	*para*-F	12.5	12.5	6.25	6.25
4	*para*-Br	12.5	12.5	6.25	25.0
5	*fluconazole*	25.0	25.0	12.5	25.0
